# Factors Determining Sirtuin‐1 Target Engagement

**DOI:** 10.1002/adbi.202500663

**Published:** 2026-07-25

**Authors:** Petra Neumann‐Staubitz, Yannick Burgdorf, Sarah Hofmann, Heinz Neumann

**Affiliations:** ^1^ Department of Chemical Engineering and Biotechnology Darmstadt University of Applied Sciences Darmstadt Germany; ^2^ European University of Technology, European Union Darmstadt Germany

**Keywords:** lysine acylation, lysine deacetylase, sirtuins

## Abstract

Sirtuin‐1 (Sirt1) is a key NAD^+^‐dependent deacylase regulating metabolism, stress responses, genome stability, and aging. Although well‐characterized biochemically and structurally, its substrate selectivity remains unclear: in vitro, Sirt1 appears promiscuous, while in vivo it activates particular processes in response to different stimuli. Emerging evidence indicates that selectivity arises from multiple regulatory layers beyond the catalytic site. Here we review how post‐translational modifications (PTMs) ‒ including phosphorylation, acetylation, and glycosylation ‒ modulate activity, localization, and substrate affinity. For instance, phosphorylation at S27/T530 (by JNK1) or S682 (by HIPK2) affects nuclear translocation, substrate targeting, or complex formation with cofactors such as AROS and DBC1. Protein–protein interactions, for example with DBC1, PACS2, and transcription factors, further direct Sirt1 to specific substrates or compartments, functioning as allosteric regulators. Spatial compartmentalization, including nucleocytoplasmic shuttling and localization to promyelocytic leukemia nuclear bodies (PML‐NBs), integrates Sirt1 into defined signaling contexts. Moreover, liquid–liquid phase separation (LLPS) may concentrate Sirt1 and substrates within condensates, enhancing its selectivity. Overall, Sirt1 specificity emerges from PTMs, interactions, localization, and phase behavior ‒ offering a framework for developing selective modulators in metabolic and age‐related diseases.

## Graphical abstract

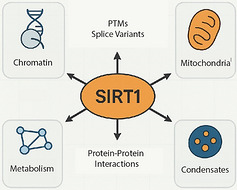

## 1 Introduction

Sirtuins constitute a highly conserved family of NAD^+^‐dependent lysine deacylases that are pivotal in regulating metabolism, stress responses, aging, and genome stability across all domains of life [1, 2, 3]. In humans, seven sirtuin isoforms (Sirt1–7) have been characterized, each exhibiting distinct localization within specific cellular compartments and demonstrating unique substrate preferences and enzymatic activities, which encompass deacetylation, desuccinylation, demalonylation, and long‐chain deacylation [4, 5]. Despite extensive characterization of the enzymatic mechanisms and structural biology of sirtuins, our understanding of the processes underlying substrate selectivity in various physiological contexts remains limited. A deeper understanding of the parameters that target sirtuins to their appropriate substrates is important for elucidating how a single enzyme can adapt to serve in so many different processes.

Traditionally, substrate specificity in sirtuins is conceptualized through the framework of direct molecular recognition, wherein residues in proximity to the active site interact with the acylated lysine and adjacent amino acids [4]. Deacylation surveys using peptide arrays have indicated minimal to no preference of Sirt1 for specific sequence contexts under in vitro conditions [6, 7, 8]. This moderate in vitro substrate preference of Sirt1 does not align with the selectivity profile observed for biochemically validated in vivo substrates [4]. Standard in vitro assays typically employ minimalist substrates (e.g., short peptides containing the modified residue) that lack the full structural context, flanking sequences, chromatin environment, and PTMs present in native substrates. As a result, they fail to capture context‐dependent regulation such as allosteric modulation, cooperative binding, or steric hindrance by partner proteins. Moreover, typical activity assays for sirtuins, such as the Fluor‐De‐Lys assay, measure catalytic turnover in isolation, often under non‐physiological conditions (e.g., high enzyme/substrate ratios, absence of co‐factors or regulators), which can mask subtle differences in substrate preference. This might explain why Sirt1 appears to have low intrinsic sequence specificity in vitro, despite clear substrate selectivity in cells.

Possible factors shaping the substrate preference of Sirt1 include spatiotemporal localization, post‐translational modifications (PTMs), alternative splicing, protein‐protein interactions, oligomerization, and phase separation [9]. In this review, we will focus on the current state of knowledge regarding the mechanisms that determine, or could determine, the substrate binding of Sirt1:

Localization of Sirt1 to different cellular compartments, such as the nucleus, the cytoplasm, or the mitochondria, and its shuttling between these is modulated in response to altered cellular conditions. The subcellular transport of Sirt1 is primarily considered in relation to two predicted NLS and NES sites, but has not been sufficiently investigated.

PTMs, such as phosphorylation, acetylation, and ubiquitination, can significantly influence the enzymatic activity, subcellular localization, and substrate affinity of Sirt1 [9, 10]. For instance, JNK1‐mediated phosphorylation of Sirt1 enhances the deacetylation of histone H3, whilst p53 remains unaffected [11]. This targeting mode is not unique to Sirt1. The yeast sirtuin Hst2 is recruited to chromatin in mitosis by phosphorylation of C‐terminal serine residues. This mark is recognized by the 14‐3‐3 protein Bmh1, which simultaneously binds to H3 S10ph, thereby juxtaposing the sirtuin to target H4 K16ac [12, 13, 14].

Protein–protein interactions represent another crucial level of substrate regulation. Sirt1 has been shown to stably associate with various substrates and co‐precipitate with numerous transcription factors, including p53 and p65, in pull‐down assays [15, 16, 17]. Proteins like DBC1 and PACS2 bind to Sirt1 and modulate its activity, possibly in different ways depending on PTMs. These interactions not only facilitate substrate targeting but may also act as allosteric regulators of sirtuin activity and specificity.

Sirt1 oligomerization is controversial: some studies suggest T530 phosphorylation promotes multimerization and regulates activity [18], while others using multiple biophysical methods show full‐length Sirt1 is monomeric in solution [19, 20, 21]. Thus, the regulation of Sirt1's in vivo substrate preference may involve the formation of transient, context‐dependent complexes or oligomers.

Finally, physical parameters such as liquid–liquid phase separation (LLPS) [22] and local concentration gradients of the co‐substrate NAD^+^ [23] are increasingly attracting attention as modulators of sirtuin activity. LLPS can concentrate enzymes and substrates within biomolecular condensates, thereby enhancing reaction kinetics and imposing selectivity by restricting the local proteome. Sirt1 has been shown to associate with phase‐separated domains such as PML bodies [15], suggesting a spatially regulated mechanism for modulating substrate accessibility.

This overview examines the factors determining the substrate selectivity of Sirt1, in particular localization mechanisms, PTMs, protein‐protein interactions, and phase separation. Understanding these factors is key to elucidating sirtuin‐dependent functions and developing therapeutic modulators.

## 2 Sirt1 Localization Beyond the Nucleus

**FIGURE 1:**
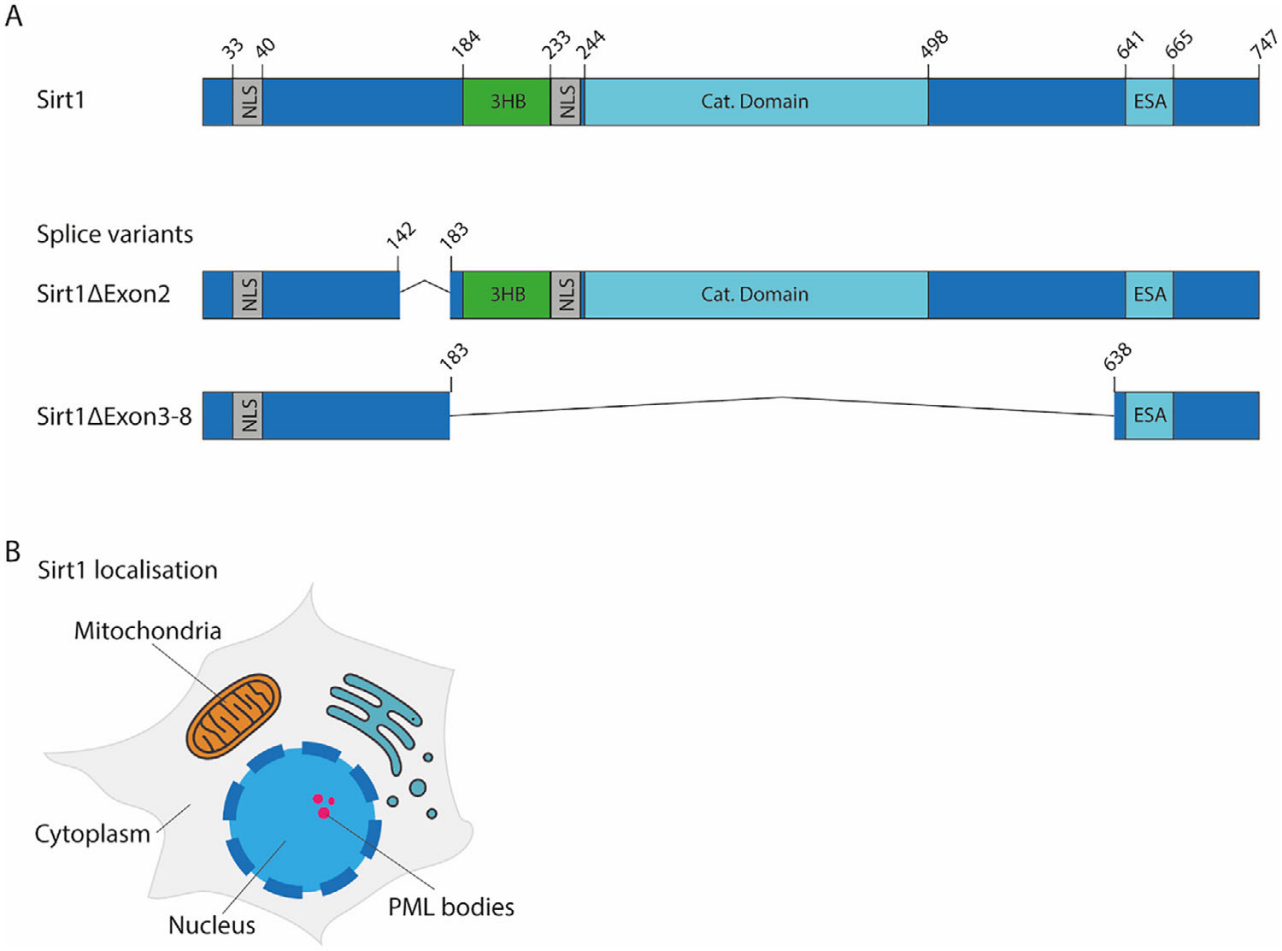
Domain structure of Sirt1, splice variants, and localization. (A) Domain structure and motifs of human Sirt1. Splice variants lacking exon 2 or exons 3–8 are shown. 3HB: three‐helix‐bundle (aa 183–233), ESA: essential for Sirt1 activity (aa 641–665, motif interacting with catalytic domain). (B) Reported subcellular localizations of Sirt1.

Spatial segregation to different subcellular locations might be a mechanism to target Sirt1 to particular substrates or processes. Sirt1 deacetylates many proteins located in the nucleus, such as histones and transcription factors. In fact, under optimal growth conditions, Sirt1 is detected in the nucleus in most cells. Sirt1 possesses two predicted NLSs and two predicted NESs [24]. The function of the two NLSs has been experimentally confirmed through the characterization of deletion and point mutants. However, analogous experiments on the two NES sites are controversial: experimentally, only NES2 had an effect on Sirt1 export to the cytosol [24], but this could be because NES2 is part of the Rossmann fold domain and a mutation could destabilize its structure. Further studies are required to define the function of NES motifs in Sirt1 localization, particularly under non‐standard conditions and in a variety of cell types (Figure 1).

There are indications that the nuclear export signal (NES) of Sirt1 is functional. Several studies have reported the nucleocytoplasmic shuttling of Sirt1 in response to changes in cellular conditions, and instances of Sirt1 localization outside the nucleus under standard culture conditions have also been documented. For example, Sirt1 is located in the cytoplasm of PC12 cells, a cell line derived from a pheochromocytoma of the adrenal medulla of rats, in both undifferentiated and differentiated cells. Using activators and inhibitors as well as siRNA of Sirt1 and by overexpressing an NLS‐deficient Sirt1 and a dominant‐negative mutant, it was shown that the cytosolic activity of Sirt1 is required for nerve growth factor (NGF)‐induced neurite growth [25]. Another publication by this group showed that Sirt1 was expressed in the cytoplasm of neuron‐like cells in the striatum of mice, while it localized to both the cytoplasm and the nucleus of ependymal cells [24].

In cardiomyocytes, the cellular localization of Sirt1 changes during differentiation: in 12.5‐day‐old mouse cardiomyocytes, Sirt1 is expressed exclusively in the nucleus, while adult cardiomyocytes express Sirt1 in both the cytoplasm and the nucleus [24]. The same authors found that Sirt1 is detected in the nucleus of C2C12 myoblast cells, but localizes in the cytoplasm following differentiation [24]. Other types of stress also influence the localization of Sirt1: in C2C12 cells, Sirt1 migrates to the nucleus in response to H_2_O_2_ stress due to JNK1 phosphorylation (at S27, S47, T530) [11]. Furthermore, in response to a differentiation stimulus, Sirt1 temporarily localizes to the nucleus in murine neural progenitor cells (NPCs), as observed in a time‐lapse experiment [26]. Using inhibitors, siRNA, a dominant‐negative Sirt1 mutant, and overexpression of Sirt1, a connection was established between the transcription factor Hes1 and Notch1: upon activation, the intracellular domain of Notch1 migrates into the cell nucleus, where it forms a complex that also includes Hes1. Hes1 normally maintains NPCs, but its concentration decreases during differentiation. The authors showed that Sirt1 suppresses Hes1 expression and consequently promotes the differentiation of NPCs into Tuj1+ neurons through the temporary localization of Sirt1 in the cell nucleus [26].

Another example of the tissue‐specific localization of Sirt1 are pancreatic islets, which belong to the endocrine pancreatic cells: Here (in mice), Sirt1 is expressed cytosolically, but not in the exocrine pancreatic tissue; in the islets, beta cells showed a faint, punctate nuclear pattern and diffuse cytoplasmic staining for Sirt1, while glucagon‐positive alpha cells showed strong, exclusively cytoplasmic staining for Sirt1 [27].

Sirt1 has also been shown to be involved in mitochondrial biogenesis, mainly through the deacetylation of PGC‐1alpha, which in turn activates genes to restore mitochondrial function. Aquilano et al. detected Sirt1 and PGC‐1alpha in the nucleus, but also in the mitochondria, particularly on the nucleoids [28]. To verify their results, they performed immunofluorescence, cross‐linking, and pull‐down assays on purified mitochondria from SH‐SY5S and HeLa cells. They hypothesize that Sirt1 and PGC‐1alpha could regulate mitochondrial genes on chromatin and mitochondrial DNA. However, they note that it is unclear how both proteins, which are known to be localized in the nucleus, can be transported into the mitochondria, as both lack a mitochondrial import sequence [28].

Sirt1 localization may also be regulated by PTMs. For example, Chattopadhyay et al. investigated the role of different glucose concentrations on the localization of Sirt1 [29]. They found that, at high glucose concentrations, Sirt1 is glycosylated at its N‐terminus within a region that is crucial for the interaction with key transcription factors like FOXO1, PPARalpha, and PGC‐1alpha. Not only was the interaction with these proteins significantly reduced, but the glycosylation also led to an active translocation of Sirt1 into the cytoplasm.

Since Sirt1's ability to move between organelles or locations within an organelle may be part of its regulation, greater emphasis should be placed on microscopic techniques that can visualize Sirt1 in different tissues under varying conditions and in different disease models. It should be noted, however, that imaging can also have its pitfalls. For example, as p53 is an important substrate of Sirt1, much of the research has focused on the role of Sirt1 in cancer cells. It has been reported that Sirt1 is mainly localized in the cytoplasm of cancer cells [30], which would explain the progression of cancer. However, researchers later demonstrated that certain antibodies and conventional cell fractionation can lead to erroneous observations. Consequently, they showed that Sirt1 is also localized in the nucleus of cancer cells [31].

It is more difficult to investigate the influence of PTMs on Sirt1 transport in vivo. In addition to substituting amino acids that mimic PTMs, using an expansion of the genetic code to insert sterically similar designer amino acids at specific sites could be a more precise way of learning more about the role of PTMs in regulating Sirt1 transport through the cell [32, 33]. Nevertheless, findings regarding Sirt1 localization outside the cell nucleus suggest that much more research is needed in this regard to understand whether various disorders and diseases result from mislocalization. Sirt1 effectors could then be developed that specifically increase its activity for particular targets [29].

## 3 Regulation of Sirt1 Substrate Selectivity by PTMs

PTMs not only regulate the localization of proteins but also modulate their substrate specificity by altering the affinity between proteins. Sirt1 is subject to a plethora of PTMs, including phosphorylation [34], acetylation [35, 36], glycosylation [29], methylation [37, 38], ubiquitinylation [39], and SUMOylation [40]. Surprisingly, relatively little research has been devoted to this type of modification of protein‐substrate interactions in Sirt1. Most of the available data on the contribution of PTMs to Sirt1 substrate binding are described in phenotypic terms rather than at a molecular or structural level. Most studies have focused on the phosphorylation of Sirt1 (with more than 30 documented phosphorylation sites [41]) and its impact on substrate selection. Here, we present selected examples of how the phosphorylation of Sirt1 regulates its substrate selectivity.

### 3.1 Effects of Severe DNA Damage on Sirt1 S682

p53 is activated by DNA damage, and deacetylation of K382 by Sirt1 suppresses its pro‐apoptotic activity. Conrad et al. demonstrated that lethal doses of the chemotherapeutic agent doxorubicin, which causes DNA damage, induce the phosphorylation of Sirt1 at S682 by HIPK2. This leads to reduced deacetylase activity toward p53, which in turn promotes apoptosis. HIPK2 has several functions within the cell, including the response to DNA damage, apoptosis, and the regulation of transcription factors. In particular, Conrad et al. found that the phosphorylation of Sirt1 occurs exclusively at lethal doses of doxorubicin and that the effect of Sirt1 phosphorylation can be prevented by HIPK2 depletion. Notably, Sirt1 colocalizes with PML‐IV within PML‐nuclear bodies (PML‐NBs), and both HIPK2 and p53 have also been identified in these structures, suggesting a potential functional interaction. Accordingly, PML deficiency abolishes the phosphorylation of Sirt1 at position S682 [42]. Furthermore, phosphorylation at S682 disrupts the interaction between Sirt1 and AROS (active regulator of Sirt1), suggesting that the deacetylating activity of Sirt1 toward p53 is reduced in the context of severe DNA damage [42].

### 3.2 Impact of Oxidative Stress on Sirt1 Phosphorylation

In co‐immunoprecipitation experiments using C2C12 cells (a mouse myoblast cell line), an interaction between Sirt1 and JNK1 (c‐Jun N‐terminal kinase 1, a member of the mitogen‐activated kinase family) has been detected, but only under conditions of oxidative stress. Under these conditions, JNK1 phosphorylates amino acid residues S27, S43, and T530 at the N‐ and C‐termini of Sirt1 and subsequently interacts with phosphorylated Sirt1 [11]. This phosphorylation‐dependent interaction results in the translocation of Sirt1 into the nucleus, a phenomenon observed exclusively in the presence of oxidative stress. Moreover, Nasrin et al. observed a shift in substrate specificity of Sirt1 as a consequence of this phosphorylation: Using a Sirt1 variant in which S27, S47, and T530 were substituted with alanine residues, a Western blot analysis of whole cell lysates demonstrated that, while the hypophosphorylated variant retained deacetylation activity toward p53, it exhibited a marked decrease in the deacetylation of histone H3. Thus, JNK‐dependent phosphorylation of Sirt1 influences not only its subcellular localization but also its substrate specificity. This represents a precedent in which the phosphorylation of Sirt1 alters its substrate specificity.

### 3.3 Impact of the Energy Master Regulator AMPK on Sirt1 Phosphorylation

AMP‐activated protein kinase (AMPK), which primarily regulates energy metabolism, has been identified on multiple occasions as an interaction partner of Sirt1, a key stress regulator linked to energy metabolism via its cofactor NAD^+^. For example, Cantó et al. demonstrated that AMPK phosphorylates peroxisome proliferator‐activated receptor gamma coactivator 1‐alpha (PGC‐1α, which is primarily involved in mitochondrial biogenesis, energy metabolism, and the stress response) in muscle cells, thereby activating Sirt1 via a mechanism that remains to be elucidated [43]. This activation leads to reduced acetylation of PGC‐1α, FOXO1 (a transcription factor that regulates glucose metabolism, insulin regulation and adipogenesis) and FOXO3a (a transcription factor involved in stress resistance, longevity, autophagy and oxidative stress), which subsequently modulate mitochondrial function. Furthermore, the deacetylation of FOXO3 by Sirt1 increases FOXO3's ability to induce cell cycle arrest and enhance resistance to oxidative stress, whilst simultaneously inhibiting its ability to induce cell death [44]. Most studies of Sirt1 phosphorylation by AMPK focused on the impact of Sirt1‐substrate interactions.

Only in a few cases have Sirt1 phosphorylation events been correlated with functional outcomes, e.g. that low expression of AMPK promotes hepatocarcinogenesis [45]. Lee et al. found that low AMPK expression promotes the development of liver cancer and identified the mechanism underlying this phenotypic observation. Ectopic expression of AMPK leads to phosphorylation of Sirt1 at position T344, thereby inactivating Sirt1. This, in turn, stabilizes p53 through increased acetylation, which subsequently promotes apoptosis. At low AMPK levels, Sirt1 is not inactivated, and apoptosis is ultimately triggered. Furthermore, they demonstrated that deacetylation activity toward p53 was impaired when a phosphomimetic form of Sirt1 (T344E) was used, whilst the T344A mutation retained deacetylation activity comparable to that of wild‐type Sirt1. Notably, all variants, including the wild type, localized to the cell nucleus, suggesting that phosphorylation at this site does not affect the localization of Sirt1 [45].

In contrast, Lau et al. independently identified the same phosphorylation site but reported the opposite functional outcome. In their study, AMPK phosphorylates Sirt1 at T344 [46], thereby releasing Sirt1 from its natural inhibitor Deleted in Breast Cancer 1 (DBC1), which consequently leads to enhanced deacetylation activity toward p53. The modulation of the interaction between DBC1 and Sirt1 was confirmed by a phosphomimetic (T344E) and by the inhibition of AMPK in cell experiments, as well as by the deletion of AMPK in MEFs, which led to an increase in p53 acetylation levels in the cellular context [46].

This discrepancy is further complicated by the structural context of T344. The residue is located within the catalytic core of Sirt1 and appears to be buried within the Rossmann fold based on available crystal structures. This raises the question of how this site would be accessible to AMPK under physiological conditions. Phosphorylation at this position would likely require significant conformational rearrangements, transient unfolding, or modification during protein folding, none of which have been experimentally demonstrated to date.

Taken together, these observations suggest that phosphorylation at T344 remains insufficiently validated and mechanistically unclear. The reliance on phosphomimetic mutants in both studies further complicates interpretation, as such substitutions do not fully recapitulate the steric and electrostatic properties of a phosphate group. It is therefore difficult to reconcile the reported functional outcomes and to assess whether T344 is a bona fide regulatory site under physiological conditions.

Future studies will be required to resolve this issue, for example, through direct detection of T344 phosphorylation under endogenous conditions by mass spectrometry, structural characterization of potential conformational states that expose this residue, and functional analyses that avoid sole reliance on phosphomimetic substitutions.

### 3.4 Convergence of Multiple Kinase Signaling Pathways in the Phosphorylation of Sirt1 T530 (Corresponding to T522 in Mice)

Sirt1 T530 is phosphorylated by JNK1 [11], CDK1/Cyclin B [34], and dual‐specificity tyrosine‐phosphorylation‐regulated kinases 1A (DYRK1A) and DYRK3 [47]. These kinases are part of different signaling pathways: JNK1 integrates multiple biochemical signals, particularly stress signals; the CDK1/Cyclin B complex is central to entry into the M phase of mitosis; DYRK1A is a kinase involved in the proliferation and differentiation of neural progenitor cells, regulates cell cycle and is required for the response to DNA‐damage; DYRK3 regulates condensate dynamics, is involved in mTORC1 signaling and regulates stress erythropoiesis. The downstream effects of the phosphorylation of Sirt1 at T530 vary considerably across these studies. While all report an increased activity of the phosphorylated form, the observed cell physiological consequences range from increased proliferation [34] and improved survival under various stress conditions [18, 47] to the control of replication origin firing [48], metabolic regulation, and differentiation during adipogenesis [49]. How can a single phosphorylation event affect such a wide variety of cellular processes? It seems likely that Sirt1‐PTMs do not act in isolation, but are rather interpreted by effector proteins in a broader context, similar to the histone code hypothesis [50] of chromatin modifications.

All these examples illustrate the influence of PTMs on the substrate selectivity of Sirt1. Future research should not only focus on other phosphorylation sites and further PTMs such as acetylation, nitrosylation, and SUMOylation, but also on the molecular mechanisms, preferably at the structural level. It should be noted that the commonly used Fluor‐de‐Lys assay employs an artificial short peptide substrate that cannot reflect many of the regulatory mechanisms discussed here. Hence, to fully understand how PTMs influence the substrate preferences of Sirt1, it is essential to assess its activity on natural substrates.

## 4 Splice Variants of Sirt1

It is generally assumed that splice variants increase protein functional diversity; however, this aspect remains poorly explored for sirtuins. All sirtuin genes contain multiple exons and are therefore likely subject to alternative splicing, a process affecting the majority of human genes [51]. Indeed, a combined bioinformatics and RT‐PCR study identified 23 isoforms across the seven human sirtuins [52]. For Sirt1, several splice variants have been reported, including Sirt1∆Exon2, which lacks important transcription factor binding sites [53], and Sirt1∆Exon3‐8, which lacks the catalytic domain [54] (Figure 1). However, most variants have been identified at the mRNA level, often supported by ectopic expression studies, while evidence for their translation into stable and functional proteins remains limited. At present, no distinct physiological function has been conclusively assigned to Sirt1 splice variants, and it remains unclear whether they contribute to cellular regulation or largely represent transcriptional noise. The discrepancy between the number of detected transcripts and the limited proteomic evidence may partly reflect methodological differences in sensitivity between sequencing and protein detection approaches [51].

Overall, the functional relevance of Sirt1 splice variants remains an open question. Future studies integrating quantitative proteomics and context‐specific analyses will be required to determine whether these variants represent functional regulators or non‐productive byproducts of gene expression.

## 5 Regulation of Sirt1 Activity by Protein–Protein Interactions

In addition to directly interacting with the acetylated lysine residue of the substrate and its immediate sequence context, Sirt1 is recruited by interactions with distal parts of the same protein or even with other subunits of a protein complex. For example, FHL2 recruits Sirt1 to FOXO1 to facilitate deacetylation of the latter [55]. Beyond targeting Sirt1 to individual proteins or lysine residues, such interactions can direct the enzymatic activity of Sirt1 toward subcellular regions or structures. Currently, more than 400 unique interactors for Sirt1 are listed in the BioGRID database [56], illustrating the potential complexity of this type of mechanism for controlling Sirt1 specificity. Here, we analyze exemplarily the interaction of Sirt1 with DBC1, which has already been studied in detail.

DBC1 is an important inhibitor of Sirt1 in mice and humans. Their interaction is regulated by cellular stress, metabolic state and PTMs, thereby controlling apoptosis, metabolism and cellular stress responses. Hence, agents that manipulate this signaling axis have potential as therapeutic strategies in metabolic and age‐related diseases. The interaction of DBC1 with Sirt1 was first observed in proteomics studies of Sirt1 co‐immunoprecipitations, and the direct nature of the interaction was demonstrated by pulldown experiments using recombinant proteins [57, 58]. Knockdown of DBC1 reduced p53 acetylation and apoptosis in response to DNA damage.

**FIGURE 2:**
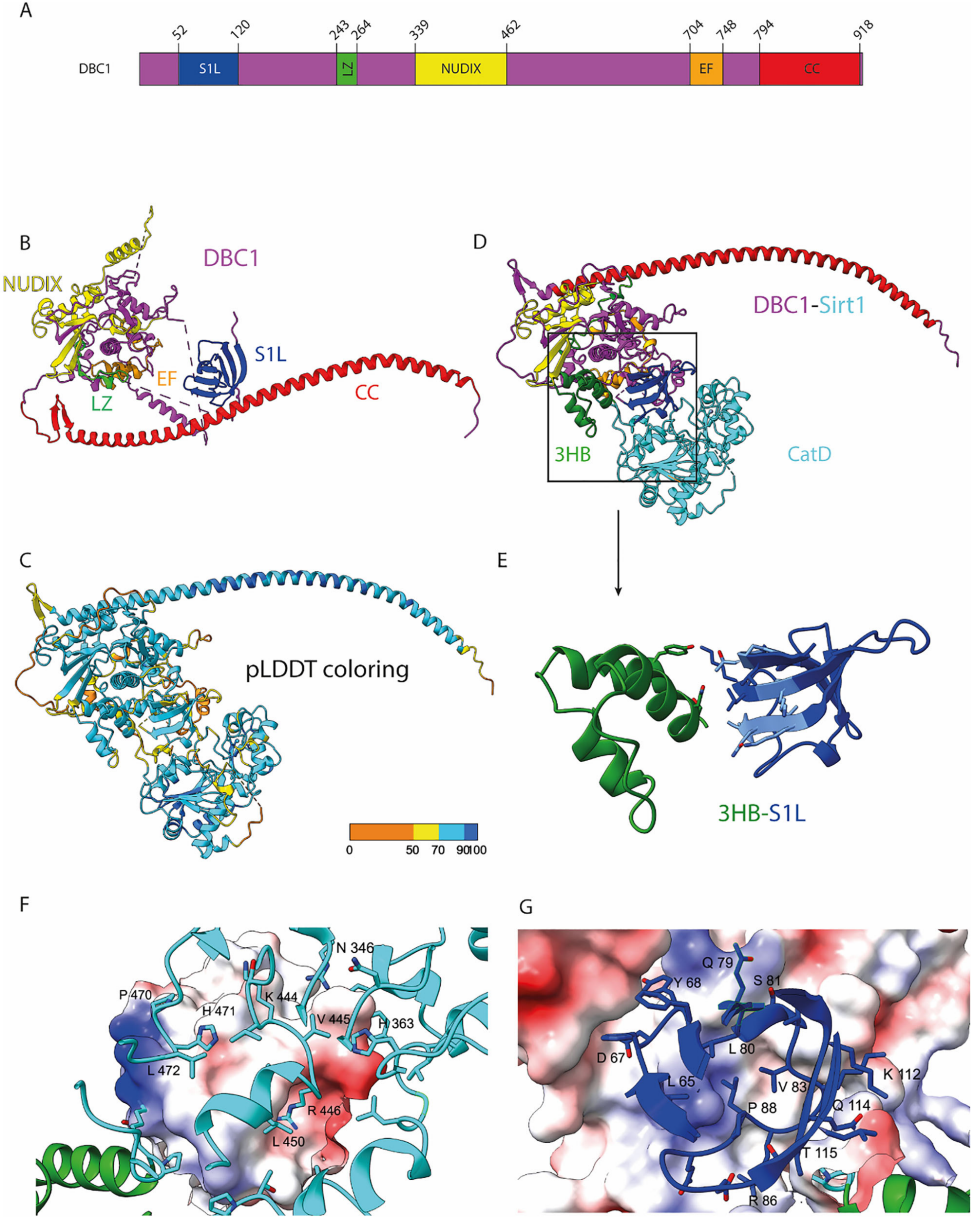
Prediction of DBC1 and DBC1‐Sirt1(Zn^2+^) complex by Alphafold3. (A) Domain structure of DBC1 predicted from sequence. (B) AF3 prediction of DBC1. (Downloaded from UniProt on May 15, 2025). (C) AF3 prediction of DBC1‐Sirt1(Zn^2+^) complex colored by pLDDT confidence score (predicted on May 15, 2025). (D) Same structure as in (C) using the color scheme as in A and B. (E) Magnified view of the interface of DBC1‐S1L and Sirt1‐3HB. (F) DBC1‐S1 interface with the catalytic domain of Sirt1. Sirt1 is depicted as a cartoon in cyan on the electrostatic surface of the S1L domain. (G) Sirt1 catalytic domain (electrostatic surface) interface with DBC1‐S1L (blue cartoons). Images created with ChimeraX 1.8.

These early studies identified a leucine zipper motif in DBC1 (aa243‐264) (Figure 2A), the deletion of which abrogated the interaction with Sirt1. This motif alone was sufficient to compete with the ESA (essential for Sirt1 activity) domain for binding to the catalytic domain of Sirt1 [59]. However, subsequent studies revealed that the N‐terminus of DBC1 contributes predominantly to the interaction, as a proteolytically truncated DBC1 lacking the first 138 amino acids, including the S1L domain, does not bind to Sirt1 [60]. Furthermore, acetylation of DBC1 at K112 within this domain reduces its ability to bind Sirt1 [61]. Moreover, phosphorylation of DBC1 at Thr454 by ATM/ATR kinases during DNA damage enhances its binding to Sirt1, thereby inhibiting Sirt1 and promoting p53 acetylation and apoptosis [62, 63].

With regard to Sirt1, it was found that the catalytic domain represents the most important interaction site, and a mutation in the catalytic histidine (H363) significantly reduced binding [58, 64]. Moreover, the DBC1‐Sirt1 interaction is disrupted by Ex527 [64], which binds to the active site of Sirt1 in the presence of the reaction product ADP‐ribose [65]. PKA‐dependent phosphorylation of Sirt1 leads to its dissociation from DBC1 in A549 and HEK293T cells [66]. Several serine residues of Sirt1 (S47, S605, and S615) were shown to be involved in this effect; however, evidence for direct phosphorylation by PKA or AMPK is missing. Later studies identified a further AMPK‐phosphorylation site at T344 within the catalytic domain of Sirt1 [46]. Mutation of this residue to glutamate disrupts the DBC1‐Sirt1 interaction. Interestingly, as part of the Rossman fold, this site is deeply hidden within the core of the catalytic domain and, judging by the crystal structure, should not be accessible to the kinase. Significant conformational changes would be required for phosphorylation to occur, and it seems unlikely that the modified enzyme would remain active. The phosphoryl group would sterically and electrostatically clash with nearby residues, particularly T260, N346, and E351.

DBC1 also interacts with the Sirt1 N‐terminus [67]. This interaction facilitates the subsequent binding of PACS2 to the three‐helix‐bundle (3HB, aa183‐233) of Sirt1. NMR spectroscopy studies demonstrated that the DBC1‐S1L domain comes into contact with several regions within the Sirt1 N‐terminus, particularly with the 3HB. It is thought that this interaction destabilizes the structure of the 3HB, thereby creating a new binding site for PACS2 [61].

All these studies suggest that the interaction between Sirt1 and DBC1 is complex. We used AlphaFold3 to generate a prediction for DBC1 (Figure 2B) and for the Sirt1(Zn^2+^)‐DBC1 complex (Figure 2C,D). We would like to point out that the predicted models are only meant to help visualize how published findings can be interpreted and that they should not be taken as evidence for the interaction per se (see Supporting Information for further details). AF3 predicts DBC1 to be a three‐domain protein, consisting of the N‐terminal S1L domain, a large middle domain, and a C‐terminal coiled‐coil domain, which are flexibly connected by long, unstructured linker regions (Figure 2B). The leucine zipper, NUDIX, and EF‐hand domains (predicted from the primary structure) are embedded within the middle domain and therefore likely do not retain their canonical functions. In the complex, which is predicted with a good confidence level, the S1L domain is in contact with the 3HB (Figure 2E) and the catalytic domain of Sirt1 (Figure 2F,G), which is consistent with biochemical and NMR spectroscopy data [61, 64]. The S1L‐3HB interface is in good agreement with NMR chemical shift perturbation data [61] and supports the model in which the binding of DBC1 leads to a conformational change in 3HB to create a binding site for PACS‐2 [67]. AF3 did not predict any interactions of DBC1 with more N‐terminal regions of Sirt1, which have been shown to contribute to complex formation [21, 61]. However, the ability of AF3 to predict interactions between intrinsically disordered regions and folded domains is currently still limited [68]. The interface between S1L and the catalytic domain of Sirt1 is extensive and dominated by hydrophobic interactions (Figure 2F,G). The prediction is consistent with the observation that mutation of residues H363 or N346 compromises the interaction [58, 64]. This might also explain why Ex527, which binds to the product complex of Sirt1 and ADP‐ribose [65], interferes with the DBC1‐Sirt1 interaction [64]. Interestingly, an AF3 prediction that includes NAD^+^ shows no interaction between the S1L domain and the catalytic domain of Sirt1. In this case, the predicted interface between the DBC1 middle domain and the catalytic domain of Sirt1 has very low confidence values.

**FIGURE 3:**
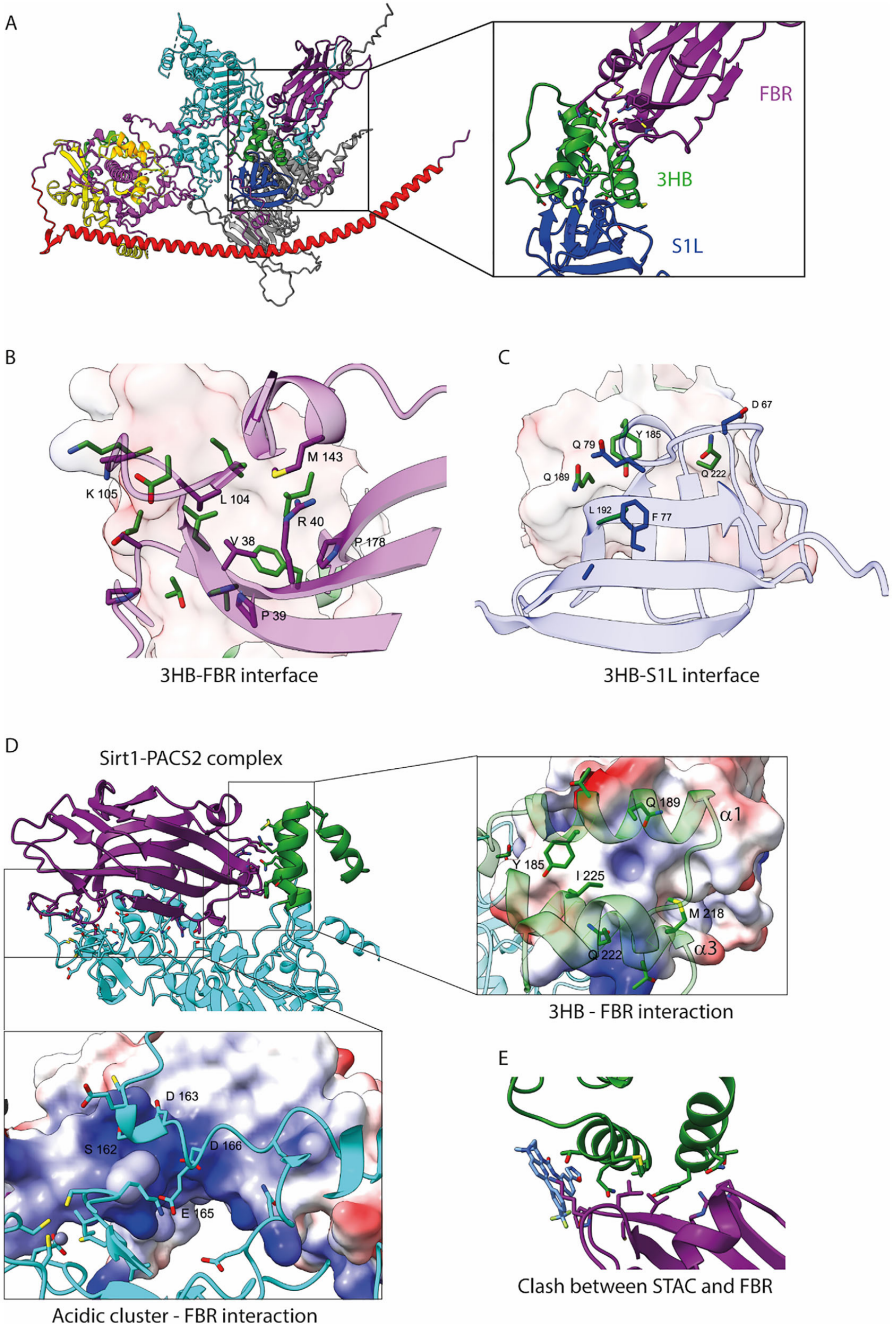
Prediction of DBC1‐Sirt1(Zn^2+^)‐PACS2 complex by Alphafold3. (A) AF3 prediction of DBC1‐Sirt1(Zn^2+^)‐PACS2 complex (predicted on May 20, 2025). Contacts with Sirt1‐3HB magnified. (B) Sirt1‐3HB (surface with interacting residues as green sticks) interface with PACS2‐FBR (purple cartoon and interacting residues). (C) Sirt1‐3HB (colored as in B) interface with DBC1‐S1L (blue cartoons and interacting residues). (D) Interaction surface of Sirt1 N‐terminus with PACS2‐FBR. Sirt1‐3HB (green cartoon and sticks) on the electrostatic surface of PACS2‐FBR (right panel). Acidic cluster of Sirt1 (cyan cartoon and sticks) in complex with electrostatic surface of PACS2‐FBR. (E) Structural alignment of the predicted complex with a STAC‐bound Sirt1 structure (cyan sticks (only STAC is shown), 4zzi [69]) shows that the STAC bound to Sirt1‐3HB would clash with the FBR domain. Images created with ChimeraX 1.8.

DBC1 has been supposed to open the structure of the Sirt1 N‐terminus to create a binding site for PACS2 [67]. The primary interaction interface on PACS2 is the N‐terminal FBR domain. We used AF3 to predict the complex of Sirt1(Zn^2+^)‐DBC1‐PACS2 (Figure 3A). In agreement with experimental observations, PACS2 interacts with Sirt1 exclusively via its FBR domain (Figure 3B). Two sites on Sirt1 interact with PACS2‐FBR: the 3HB with helices α1 and α3, and the acidic cluster (aa161‐166) (Figure 3D). Both motifs have been shown previously to contribute to complex formation [67]. It is assumed that STACs disrupt the binding of PACS2 by stabilizing the 3HB and thereby blocking the interaction with helix α3 [67]. In an overlay of the predicted structure with a STAC‐bound Sirt1 structure (4zzi, [69]), binding of the STAC and PACS2 to Sirt1 3HB would, for steric reasons, be mutually exclusive (Figure 3E). Interestingly, DBC1 S1L continues to bind to the Sirt1 3HB via a different interface compared to the binary complex (Figure 3C).

As outlined above, data from biochemical, structural, and theoretical studies indicate that the interaction between DBC1 and PACS‐2 with Sirt1 is very complex. The prediction that DBC1 engages with the catalytic domain of Sirt1 and thereby blocks substrate binding is consistent with the general view that DBC1 negatively regulates Sirt1 activity. Most studies have investigated the impact of DBC1 on p53 acetylation and the downstream processes affected by this, such as DNA repair and apoptosis [57, 58, 62]. The deacetylation of other transcription factors is regulated in a similar manner by DBC1: H_2_O_2_‐stimulated FOXO3 acetylation is increased in HEK cells upon DBC1 overexpression [58], whereas PGC‐1α acetylation is reduced in PACS‐2 knockout mice [67]. However, it is conceivable that certain substrate proteins may interact with the DBC1‐Sirt1 complex in a way that exposes the substrate binding pocket. By “hitchhiking” on DBC1, which contains an NLS [70], Sirt1 might shuttle back and forth between the cytosol and the nucleoplasm [64]. Alternatively, writers of PTMs, non‐coding RNAs or small molecules such as STACs, which interfere with the Sirt1‐DBC1 interaction, might accumulate in subcellular compartments and direct Sirt1 activity toward particular substrates and pathways by releasing it from inhibition.

## 6 Sirt1 Oligomerization

According to the Protein Data Bank (PDB), the majority of proteins form homo‐oligomers, which are thought to play a regulatory role in complex formation and to confer specificity and diversity on protein‐protein interactions [71]. Several studies have addressed whether sirtuins are regulated by oligomerization: in yeast Hst2 (homologous to human Sirt2), the formation of homotrimers was observed in a crystal structure and in sedimentation experiments (with an affinity in the low to mid‐micromolar range) [72] and in human Sirt1 in a size exclusion analysis [73].

Guo et al. found that T522 in mice (T530 in human Sirt1) becomes phosphorylated in response to heat shock in HEK293T Sirt1 knockdown cells [18]. Using Sirt1 point mutants (T522A and T522E) in light scattering and cryo‐EM negative staining experiments, the authors found that Sirt1 T522E is monomeric, whereas T522A forms multimers and aggregates. A deacetylation assay using p53 K382ac as substrate showed that all three recombinant proteins deacetylated p53 at low p53Kac concentrations, but Sirt1 T522E deacetylated p53 significantly more efficiently at high substrate concentration. The authors hypothesized that T522, located in a hinge region between the catalytic core domain and the C‐terminus, alters the conformation of the disordered N‐ and C‐terminal regions of Sirt1, thereby altering their inhibitory impact on Sirt1 activity. Notably, these observations apply only to the Sirt1 T522E mutant in mice.

Lakshminarasimhan et al. also examined the capability of Sirt1 to form oligomers [19]. They assumed that the N‐ and C‐terminal extensions of Sirt1 in particular drive oligomerization, and therefore worked with full‐length Sirt1 as well as truncated proteins. Sirt1 did indeed behave like a trimeric protein complex in size‐exclusion chromatography (SEC). However, as SEC results can be misleading, particularly when proteins such as the full‐length Sirt1 do not have a spherical shape, they carried out further experiments, including EM negative stain, analytical ultracentrifugation, and CD spectroscopy, and found that the full‐length Sirt1 protein exists as a monomer in solution. In contrast to the other publications mentioned, the latter study clearly characterized the monomeric state of Sirt1 in solution biochemically, which does not rule out PTM‐mediated oligomerization in the cellular context.

Hence, oligomerization of Sirt1 cannot be recapitulated in simplified biochemical conditions but may still contribute to its regulation in vivo in the presence of additional modifications and factors.

## 7 Phase Separation

The formation of membrane‐less organelles is known as liquid‐liquid phase separation (LLPS) and has been the subject of increasing research over the past decade. In LLPS, multiple low‐affinity interactions between biomolecules cause them to transition into a new phase, leaving behind a diluted surrounding medium [74]. These properties are attributed to the intrinsically disordered regions (IDRs) of some proteins [75], which are also present in Sirt1 [20]. However, globular proteins lacking IDRs have also been observed to undergo LLPS by hydrophobic interactions, pi‐pi or pi‐cation interactions, electrostatic interactions, or hydrogen bonding [76, 77]. LLPS is characterized by stable, liquid‐like droplets in a relatively homogeneous surrounding medium. These condensates harbor unique local chemical environments whilst simultaneously allowing the exchange of various molecules with the surrounding medium. These levels of organization seem to play an important role in enabling and coordinating the many complex biochemical reactions and processes that take place within the cell [78]. Phase separation could provide a mechanism to spatially and temporally concentrate Sirt1 with substrates or interacting partners of specific cellular pathways, especially since Sirt1 has a broad substrate spectrum [8]. Alternatively, phase separation could drive proteins into a depository body, rendering them inactive to substrates in the immediate vicinity.

### 7.1 Sirt1 Colocalizes to PML‐NBs

The earliest connection between Sirt1 and LLPS was made in promyelocytic leukemic nuclear bodies (PML‐NBs) by Langley and coworkers [15]. PML‐NBs are nuclear membrane‐less organelles that play a role in numerous cellular processes such as protein modification, transcriptional regulation, and DNA damage response, repair, and apoptosis [79]. They are comprised primarily of the tumor‐suppressor protein PML, which self‐assembles into a shell‐like structure that encloses various recruited PML‐associated proteins in its core [80]. Sirt1 was identified as one of these PML‐associated proteins, recruited to PML‐NBs via PML isoform IV [15]. Suspecting p53 to be a substrate of Sirt1, Langley et al. showed that PML IV proteins, Sirt1, and p53 all co‐localized in PML‐NBs [15]. As Langley and coworkers suggest, PML‐NBs appear to function as organizing centers that bring Sirt1 and its substrate p53 together. In this respect, Sirt1 can be considered a client protein of PML‐NB condensates.

As mentioned earlier, Conrad et al. identified a regulatory mechanism governing Sirt1 activity within PML‐NBs. They found that the tumor suppressor homeodomain‐interacting protein kinase 2 (HIPK2), a DNA damage‐responsive kinase, co‐localized with Sirt1 at PML‐NBs. Within the condensate, HIPK2 phosphorylates Sirt1 at Ser682, which inactivates its deacetylase activity, likely by impairing its binding to AROS. Thus, efficient p53 activation in response to DNA damage could be facilitated, further supporting the role of PML‐NBs as organizing centers for cellular processes [42].

Langley et al. also hypothesized that Sirt1 might be involved in the regulation of the PML IV protein and thus in the formation of PML‐NBs ‒ an assumption that was later confirmed by Campagna and coworkers [81]. They found that Sirt1 positively regulates PML IV protein levels and PML‐NB formation by promoting the SUMOylation of PML IV, and that this occurs independently of Sirt1's deacetylase activity. However, there are also other deacetylases that may have rescued the lack of Sirt1 activity. In fact, Guan et al. later showed that the PML IV protein must first be deacetylated before it can be SUMOylated [82]. This process is mediated not only by Sirt1 but also by Sirt5, with both being translocated from the cytosol to the nucleus in parallel with the PML IV protein under oxidative stress. Nuclear Sirt1/Sirt5 deacetylation of the PML IV protein occurs specifically at position K487, a site within the functional nuclear localization sequence (NLS) of the PML IV protein. The acetyl group would otherwise block an essential SUMOylation site at K490, which is necessary for its incorporation into PML‐NBs [82].

How is Sirt1 recruited by PML proteins? It is thought that the self‐association of PML proteins is triggered by the interaction of their N‐terminal RBCC/TRIM motifs [80]. Following SUMOylation, the SUMOylated PML protein can undergo non‐covalent interactions with other PML proteins via its SUMO‐interacting motif (SIM, ^508^VVVI), thereby stabilizing its shell‐like structure [83]. At the same time, the SIM serves as an interaction site for other SUMOylated proteins, thereby providing a mechanism for the recruitment of client proteins [84]. Indeed, Sirt1 contains a SUMOylation site within the IDR of its C‐terminus at K734 [40], and it is tempting to speculate that it is recruited to the PML protein via a SUMO‐SIM interaction (see Supporting Information for further details), but this still needs to be tested experimentally.

### 7.2 Chromatin: Sirt1 Is Essential for Chromosome Condensation in Mitosis

Chromosomal liquid‐liquid phase separation during mitosis is crucial for the organization and function of the cell. A key step in early mitosis for the correct segregation of chromosomes is chromatin condensation [85]. This process is driven by condensins and histone‐histone interactions, in which Sirt1 plays a central role [48, 86, 87, 88].

Condensins help condense the DNA into chromosomes by reeling in the chromatin chain to extrude a loop [89]. This requires the loading of condensin onto chromatin [90]. While the mechanistic process has not yet been fully elucidated, Fatoba et al. showed that Sirt1 is essential for condensin I loading onto chromatin and for correctly condensed, organized and aligned chromosomes in mitosis [86]. In addition to condensins, neighboring histone‐histone interactions are also necessary for chromatin condensation. These interactions are driven by the deacetylation of histones, mediated by Sirt1, among others. For example, in human cells, Sirt1 is involved in the deacetylation of H3K9 or H4K16 at the onset of prophase [87]. In yeast, however, the sirtuin Hst2 is recruited to chromatin via phosphorylation of histone H3 S10 to deacetylate H4 K16ac [12, 13, 14].

Sirt1 is recruited to chromatin through interaction with various chromatin‐associated proteins. Utani et al. found that Sirt1 T530ph associates with chromatin during prophase/prometaphase, is released from mitotic chromosomes during metaphase, and re‐associates with chromatin during cytokinesis in HCT116 cells [48]. The elevated levels of chromatin‐bound Sirt1 T530ph during the G1 and G2 phases relative to the S phase, along with its enrichment at replication origins (and, to a lesser extent, at replication forks) suggest that Sirt1 T530ph preferentially associates with DNA replication origins and interacts with components of pre‐replication complexes as well as with replication fork proteins.

The physical basis of the chromatin phase separation process and the role of Sirt1 in this process have not yet been fully explained, as many factors influence chromatin structure and organization, such as chromatin‐associated proteins, various histone modifications and gene activity patterns, and the presence of macromolecules and cations [85, 91, 92]. We know that chromatin condensation is a highly dynamic process and that Sirt1 activity is one of many essential components driving chromatin condensation in mitosis.

So far, Sirt1 has rarely been mentioned in connection with condensates in cells. One can only speculate as to the reasons for this: perhaps, under the cellular conditions studied, PML bodies with Sirt1 do not form. Or the proportion of endogenous Sirt1 condensates is too low to be detected using standard methods. The use of high‐content microscopy, which generates vast datasets and allows different cell populations to be analyzed using neural networks, would help in the future to identify structures within a cell that are overlooked by conventional imaging.

## 8 Conclusions

Sirt1 exemplifies how enzyme specificity can be achieved through multilayered regulation rather than strict sequence recognition. Its apparent substrate promiscuity in vitro contrasts with tightly controlled activity in vivo, orchestrated by a complex network of post‐translational modifications, interaction partners, splice variants, multimerization, phase separation and spatial compartmentalization. Phosphorylation and other PTMs dynamically modulate Sirt1's localization and interaction interfaces, while cofactors such as DBC1, PACS2, and AROS fine‐tune its activity toward defined substrates. Emerging evidence for liquid–liquid phase separation and recruitment to biomolecular condensates adds a new dimension to our understanding of Sirt1 regulation, suggesting that physical reorganization within the cell can dictate enzymatic function. Together, these mechanisms highlight Sirt1 as a context‐dependent signaling hub that integrates metabolic signals and stress responses.

Despite significant progress, key questions remain unresolved. A central challenge is to understand how the multiple regulatory layers acting on Sirt1 are integrated in physiologically relevant contexts such as metabolic stress, DNA damage response, and aging. In particular, it remains unclear how combinations of post‐translational modifications, interaction partners, and subcellular localization collectively determine substrate selection in vivo. Addressing these questions will require more quantitative and context‐resolved approaches. Emerging technologies such as single‐cell proteomics, high‐content imaging, and advanced structural methods capable of capturing conformational dynamics are likely to provide important insights. In addition, approaches that enable controlled perturbation of Sirt1 interactions in living cells, including optogenetic and chemical biology tools, may help disentangle causal relationships within this regulatory network. Finally, a better mechanistic understanding of Sirt1 regulation will be essential to guide therapeutic strategies aimed at selectively modulating its activity without perturbing its broader physiological functions.

## Conflicts of Interest

The authors declare no existing conflict of interest.

## Supporting information




**Supporting File**: adbi70140‐sup‐0001‐SuppMat.docx.

## Data Availability

The authors have nothing to report.
