# Supplementary material for: Factors Determining Sirtuin‐1 Target Engagement
Source: Adv Biol (Weinh). 2026 Jul 25;10(7):e00663. doi: 10.1002/adbi.202500663 (PMC13401226; doi:10.1002/adbi.202500663)
Supplement: Supplementary file 1 — Supporting File: adbi70140‐sup‐0001‐SuppMat.docx. [file ADBI-10-e00663-s001.docx]

**Supporting Information**

**Factors determining Sirtuin-1 target engagement**

Petra Neumann-Staubitz^1,2^, Yannick Burgdorf^1,2^, Sarah Hofmann^1,2^ and Heinz Neumann^1,2,*^

^1^Department of Chemical Engineering and Biotechnology, Darmstadt University of Applied Sciences, Stephanstrasse 7, 64295 Darmstadt, Germany

^2^European University of Technology, European Union

*Corresponding Author: Heinz Neumann

email: heinz.neumann@h-da.de

tel.: +49 6151 533 68 203

**Methodology and Interpretation of AlphaFold3 Predictions**

**1. Purpose of the Predictions**

The AlphaFold3 (AF3) predictions presented in the main text were **not intended as primary research or high-resolution structural models**. Instead, they serve as **integrative, hypothesis-generating tools** to visualize how existing experimental data ‒ such as mutagenesis studies, NMR chemical shift perturbations, co-immunoprecipitation assays, and functional readouts ‒ can be interpreted within a structural framework.

**2. Input Data and Model Generation**

All predictions were generated using **AlphaFold3** on the AlphaFold Server **(**[**https://alphafoldserver.com/**](https://alphafoldserver.com/)**)**  with the following inputs:

- **Protein sequences (all H. sapiens)**:
  - Sirt1 (UniProt: Q96EB6), full-length protein (aa 1 – 747, with PTMs where indicated) including a Zn^2+^-ion.
  - DBC1 (UniProt: Q8N163), full-length (aa 1 – 923).
  - PACS2 (UniProt: Q86VP3), full-length (aa 1 – 889).
  - PML-IV (UniProt: P29590-5), aa 1 – 633, including five Zn^2+^-ions.
  - SUMO1 (UniProt: P63165), aa 1 – 101.
- **Complexes modeled**:
- **Sirt1(Zn²⁺)-DBC1** (binary complex)
- **Sirt1(Zn²⁺)-DBC1-PACS2** (ternary complex)
- **Sirt1(Zn²⁺)-DBC1 with NAD⁺** (to assess ligand effects)
- **Sirt1** **S682ph(Zn²⁺)-PML-IV(6xZn^2+^)-SUMO** (to assess Sirt1-SUMO interaction with PML-IV)
- **Model building**:
  - Default AF3 settings [1].
  - Confidence scores (pLDDT) were used to assess local quality of the models. Global confidence scores (ipTM, pTM) were low because of the presence of large unstructured regions.
  - Models were imported in ChimeraX 1.8 [2] to generate TIFF-images.

**3. Interpretation Framework: How to Read the Models**

- **Purpose of the AF3 predictions:**
- Predict **plausible spatial arrangements** of folded domains.
- Identify **potential interaction interfaces** consistent with mutagenesis data (e.g., H363, N346, T344).
- Highlight **steric incompatibilities** (e.g., between STACs and PACS2 binding to the 3HB).
- Provide **hypotheses for functional mechanisms** (e.g., DBC1-induced destabilization of the 3HB).
- **Limitations:**
- **Intrinsically disordered regions (IDRs)** (e.g., linker regions in DBC1 or the N-terminus of Sirt1) and their interactions are predicted with very low confidence and are usually omitted from the presented images.
- **Dynamic or transient interactions** (e.g., phosphorylation-induced conformational changes) are not resolved in the predicted models.
- Models do not provide **atomic-level accuracy** for flexible or low-confidence regions.
- **No refinement**: Models were **not refined** using experimental data (e.g., cryo-EM density maps, Rosetta, or Phenix).

We emphasize that these models are **speculative, illustrative, and context-dependent**. They should **not be interpreted as definitive structures**, but rather as **plausible frameworks** that align with current experimental knowledge.

**Alphafold3 prediction of PML-IV – Sirt1 – SUMO complex**

To at least address this possibility, we have used Alphafold3 [1] to generate a prediction between the interaction of Sirt1 S682ph(Zn^2+^)-SUMO with PML-IV(6xZn^2+^). Since AF3 currently does not include SUMO as a PTM, we entered Sirt1 S682ph(Zn^2+^) and SUMO as separate proteins to predict the interaction with PML-IV. Despite AF3 prediction with lower confidence level, the model shows the interaction and parallel conformation of the PML-IV-SIM (residues 555-599) with the β2-strand of SUMO (residues 35-37) and the burying of PML-IV-SIM V557 and I559 into the hydrophobic groove formed by the β2-strand and the α1-helix of SUMO1 (Figure S1A and B), which is in agreement to NMR spectroscopy data [3,4]. Although Sirt1 K734 is not directly conjugated to the C-terminus of SUMO, the pronounced conformational flexibility of the C-terminal regions in both SUMO1 [5] and Sirt1 [6] likely allow the complex, as depicted, to form. In addition, the model reveals an interesting interaction interface between the three-helix bundle (3HB, aa183-233) of Sirt1 and the B2-box of PML-IV. Helices α1 and α3 of Sirt1 form a small hydrophobic cleft, into which L217 of PML-IV protrudes to interact with I225 and V188 of Sirt1. Outside this cleft, Tyr185 of Sirt1 undergoes hydrophobic interactions with A216 of PML-IV, while Q189 and Q222 of Sirt1 form hydrogen bonds with S223 and S214 of PML-IV, respectively (Figure 1A). Intriguingly, this binding interface is approximately 120° from the helix-turn-helix (α2-T-α3) binding interface for small molecule sirtuin-activating compounds (STACs) [7].


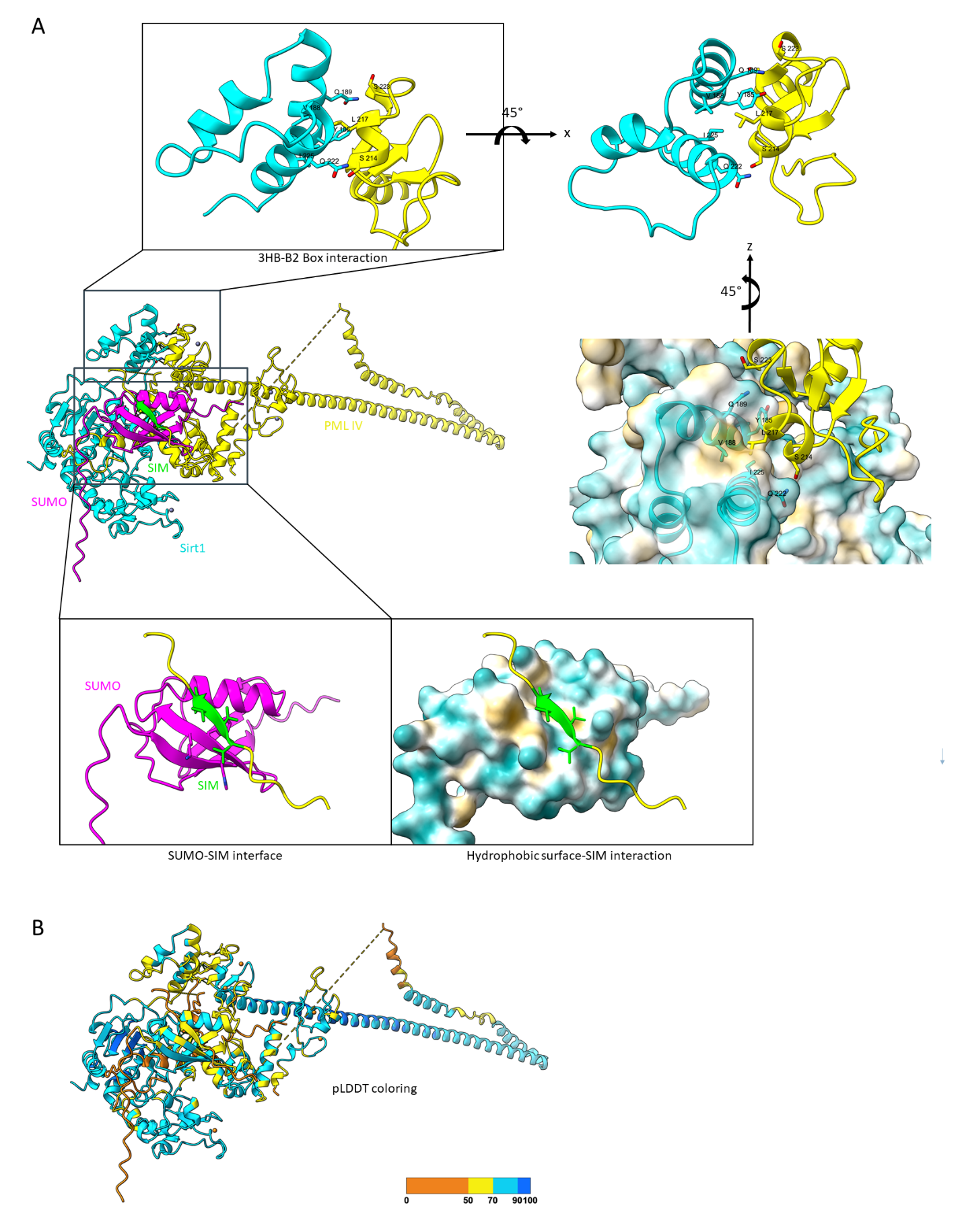


**Figure S1:** Prediction of Sirt1 S682ph(Zn^2+^)-SUMO-PML-IV(6xZn^2+^) complex by Alphafold3. A) AF3 prediction of Sirt1 S682ph(Zn^2+^)-SUMO-PML-IV(6xZn^2+^) complex (predicted on October 7, 2025). Contacts of Sirt1-PML-IV and SUMO-SIM magnified. Sirt1 depicted as cartoons in cyan, PML-IV in yellow with SIM-motif in green and SUMO in magenta. B) AF3 prediction of Sirt1 S682ph(Zn^2+^)-SUMO-PML-IV(6xZn^2+^) complex coloured in pLDDT confidence score (predicted on October 7, 2025). Images created with ChimeraX 1.8.

**References**

[1] Abramson, J. *et al.* (2024). Accurate structure prediction of biomolecular interactions with AlphaFold 3. *Nature*. https://doi.org/10.1038/s41586-024-07487-w.

[2] Pettersen, E.F. *et al.* (2021). UCSF ChimeraX: Structure visualization for researchers, educators, and developers. *Protein science : a publication of the Protein Society*. https://doi.org/10.1002/pro.3943.

[3] Lussier-Price, M. *et al.* (2022). Zinc controls PML nuclear body formation through regulation of a paralog specific auto-inhibition in SUMO1. *Nucleic acids research*. https://doi.org/10.1093/nar/gkac620.

[4] Hecker, C.-M. *et al.* (2006). Specification of SUMO1- and SUMO2-interacting Motifs*. *Journal of Biological Chemistry*. https://doi.org/https://doi.org/10.1074/jbc.M512757200.

[5] Macauley, M.S. *et al.* (2004). Structural and Dynamic Independence of Isopeptide-linked RanGAP1 and SUMO-1 *. *Journal of Biological Chemistry*. https://doi.org/10.1074/jbc.M408705200.

[6] Davenport, A.M. *et al.* (2014). Structural and functional analysis of human SIRT1. *Journal of Molecular Biology*. https://doi.org/10.1016/j.jmb.2013.10.009.

[7] Dai, H. *et al.* (2015). Crystallographic structure of a small molecule SIRT1 activator-enzyme complex. *Nature Communications*. https://doi.org/10.1038/ncomms8645.
